# Effectiveness and Safety of Portable Ultrasound-Guided Pharmacopuncture for Cervical Myofascial Pain Syndrome: A Prospective Observational Multi-Center Study

**DOI:** 10.3390/medicina61081371

**Published:** 2025-07-29

**Authors:** Robin Kwon, Kwangho Kim, Young-Ung Lee, Sanghyuk Kwon, Juhwan Song, Seongjun Park, Junhui Kwon, Hyeon Joon Hong, Youngyun Lee, Jungtae Leem, Hongmin Chu, Cheol-Hyun Kim

**Affiliations:** 1 The Academy of Korean Medicine Clinical Anatomy, Seoul 03183, Republic of Korea; bean5@naver.com (R.K.); bzkimkh@naver.com (K.K.); www8744@naver.com (Y.-U.L.); shyuk0817@naver.com (S.K.); jhsong1035@naver.com (J.S.); park.seongjun.4@gmail.com (S.P.); ahqualia@gmail.com (J.K.); ga99193@naver.com (H.J.H.); betteryy2@naver.com (Y.L.); 2 Gangdong Forest Hospital of Korean Medicine, Seoul 05398, Republic of Korea; 3 Department of Korean Medicine, Jinjeop Hanyang Hospital, Namyangju 12067, Republic of Korea; 4 Mullae Majubom Korean Medicine Clinic, Seoul 07281, Republic of Korea; 5 Anjung Korean Medicine Clinic, Seoul 04173, Republic of Korea; 6 Gwanghwamun Kyung Hee Korean Medicine Clinic, Seoul 03183, Republic of Korea; 7 Kangnyung Korean Medicine Clinic, Anyang 13939, Republic of Korea; 8 Research Center of Traditional Korean Medicine, College of Korean Medicine, Wonkwang University, Iksan 54538, Republic of Korea; 9 Department of Il-won Integrated Medicine, Wonkwang University Korean Medicine Hospital, Iksan 54538, Republic of Korea; 10 Department of Diagnostics, College of Korean Medicine, Wonkwang University, Iksan 54538, Republic of Korea; 11 Mapo Hongik Korean Medicine Clinic, Seoul 04173, Republic of Korea; 12 Korean Medicine Convergence Research Information Center for Stroke, College of Korean Medicine, Wonkwang University, Gwangju 61729, Republic of Korea; 13 Department of Internal Medicine, College of Korean Medicine, Wonkwang University, Iksan 54538, Republic of Korea

**Keywords:** Korean medicine, cervical myofascial pain syndrome, pharmacopuncture, acupuncture, ultrasound-guided

## Abstract

*Background and Objectives*: This study aimed to evaluate the clinical effectiveness and safety of ultrasound-guided pharmacopuncture (UGP) in comparison to non-guided pharmacopuncture (NGP) for the treatment of acute cervical myofascial pain syndrome (C-MPS) in primary care settings. *Materials and Methods*: This multi-center, prospective observational study included 97 patients diagnosed with acute C-MPS. Participants received a single session of either UGP or NGP at one of seven primary care institutions. Pain intensity was measured using the Numerical Rating Scale (NRS), and cervical function was assessed through active Range of Motion (ROM) tests conducted before and after treatment. We conducted follow-up interviews within 48 h after treatment to monitor adverse events. *Results*: Both groups showed significant improvements in pain levels and cervical ROM after treatment. The UGP group showed a greater reduction in NRS scores compared to the NGP group (*p* < 0.001). Notable enhancements in cervical extension and rotation on the affected side were also observed in the UGP group (*p* < 0.01), whereas changes in flexion and lateral flexion were similar between the two groups. No serious adverse events were reported. *Conclusions*: UGP has shown superior pain reduction and a greater improvement in specific cervical motions compared to non-guided treatments, indicating enhanced precision and therapeutic efficacy. Furthermore, no serious adverse events were reported, suggesting that UGP is a safe and effective non-surgical intervention for acute C-MPS in real-world primary care settings.

## 1. Introduction

Myofascial pain syndrome (MPS) is a soft tissue pain disorder characterized by localized and referred pain originating from trigger points (TrPs) within muscles or fascia. It is among the most common causes of both acute and chronic musculoskeletal pain, particularly in individuals exposed to prolonged static postures or repetitive strain, such as office workers and healthcare professionals [1,2,3,4]. Although the exact pathophysiology of MPS remains unclear [5], factors such as trauma, postural imbalance, and psychological stress are believed to contribute to the development of TrPs [3].

Cervical myofascial pain syndrome (C-MPS), a subtype of MPS, is one of the leading causes of chronic neck pain [6]. TrPs in muscles such as the levator scapulae, upper trapezius, and infraspinatus not only generate localized discomfort but also cause radiating pain to the head and upper limbs [6,7,8,9]. These patterns may contribute to secondary musculoskeletal issues, including altered posture and referred neuropathic symptoms [5].

Musculoskeletal pain disorders encompass more than just physical discomfort; they significantly diminish quality of life, impair occupational performance, and lead to increased utilization of healthcare resources [10]. According to the World Health Organization (WHO) and the Global Burden of Disease study, musculoskeletal pain consistently ranks among the leading contributors to Disability-Adjusted Life Years (DALYs) worldwide [11]. In the United States, over 100 million adults report experiencing chronic pain, with MPS and fibromyalgia identified as two of the most common underlying causes [6]. Furthermore, in 2017, the global age-standardized prevalence and incidence rates of neck pain were reported as 3551.1 and 806.6 per 100,000 population, respectively, highlighting the significant global burden of this condition [12,13]. Similarly, in Republic of Korea, the Health Insurance Review and Assessment Service (HIRA) data from 2023 revealed that dorsalgia (M54, KCD-8), which includes cervicalgia (M54.2), ranked first in outpatient visits and reimbursement costs in Korean medicine services [14]. These findings highlight the pressing need for effective and cost-efficient treatment strategies.

Reflecting this trend, clinical interest in C-MPS has been steadily increasing within the field of Korean medicine, accompanied by various non-surgical treatment approaches. A wide range of therapeutic modalities—including acupuncture, acupotomy, pharmacopuncture, and chuna manual therapy—has been utilized for C-MPS [15]. More recently, ultrasound-guided acupuncture (UGA) has emerged as a promising alternative [16,17,18], and ultrasound-guided pharmacopuncture (UGP) combines the therapeutic principles of pharmacopuncture treatment with the anatomical precision of real-time ultrasound imaging, allowing for accurate delivery of injectates into deep or complex interfascial spaces such as the SLS triangle (the interfascial space formed by the Splenius Capitis, Levator Scapulae, and Serratus Posterior Superior) [7,8,19]. Early studies, including case reports and small trials, suggest that UGP improves pain and range of motion while minimizing procedural risks [7,8,19].

However, most available evidence remains anecdotal or observational. Accordingly, this study was conducted as a multi-center, prospective observational study across seven Korean Medicine institutions, with the aim of objectively evaluating the clinical effectiveness and safety of UGP. We hypothesized that UGP would result in greater pain relief and functional improvement, with fewer adverse events, compared to non-guided pharmacopuncture for patients with acute C-MPS. This research serves as a follow-up to a previously published study protocol and is intended to provide foundational data for designing future randomized controlled trials (RCTs) [8].

## 2. Materials and Methods

### 2.1. Study Design

This study was designed as a prospective observational study conducted in primary care clinics to evaluate the therapeutic effectiveness and safety of UGP in patients with acute C-MPS who present with neck and shoulder pain.

UGP was performed by identifying the interfascial space formed by the Splenius Capitis (SC), Levator Scapulae (LS), and Serratus Posterior Superior (SPS) muscles—referred to as the SLS triangle—under ultrasound guidance, followed by the injection of Hominis Placenta (HP) pharmacopuncture solution into the targeted area.

Participants were assigned to either the UGP group or the non-guided pharmacopuncture (NGP) group using a site-based allocation method, in which each institution recruited participants for only one of the two groups.

All participants received a single treatment session, and evaluations were conducted both before and after the procedure. These evaluations included pain assessment using the Numerical Rating Scale (NRS) and cervical function assessment using the Range of Motion (ROM) measurement. We monitored adverse events through a follow-up telephone interview conducted 24 to 36 h post-treatment.

In both groups, HP pharmacopuncture solution was used as the sole intervention. In the UGP group, the practitioner monitored the ultrasound screen in real time and injected the pharmacopuncture solution into the targeted area within the SLS triangle. During the procedure, the ultrasound screen was shielded from the patient to prevent visual access. In contrast, in the NGP group, the practitioner estimated the location of the SLS triangle through palpation, positioned the ultrasound probe over the area, and administered the pharmacopuncture treatment without viewing the ultrasound image. In this group, neither the practitioner nor the patient had access to the ultrasound screen.

The design of this study was based on the methodology proposed by Kim Jong Uk et al. [20]. More detailed information on the study design and intervention protocol can be found in our previously published protocol paper (Kim et al. [8]).

**Figure 1:**
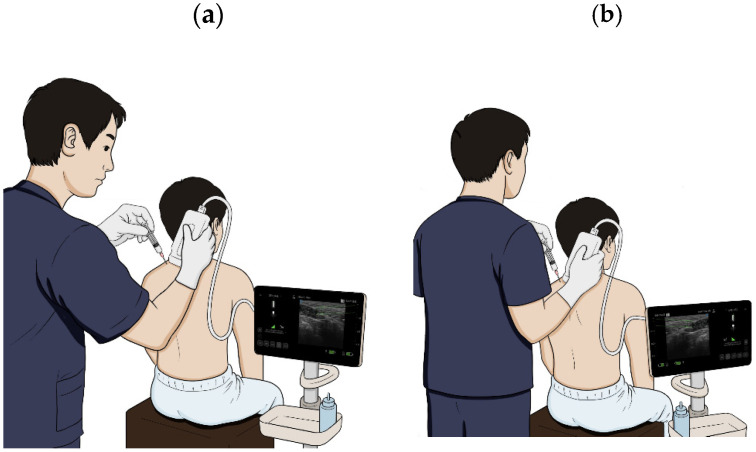
Procedure settings in ultrasound-guided pharmacopuncture (UGP) and non-guided pharmacopuncture (NGP) groups: (**a**) In the UGP group, the practitioner performs the treatment while observing the ultrasound monitor, while the patient’s view of the screen is intentionally blocked. (**b**) In the NGP group, the procedure is performed without visual reference to the ultrasound screen by either the practitioner or the patient.

An overview of the study procedure and flow is summarized in Figure 1.

### 2.2. Ethical Considerations

The study was initially designed to implement a crossover approach, in which each participating institution would alternate between conducting UGP and NGP during different periods. This strategy aimed to minimize inter-institutional bias. However, the Institutional Review Board (IRB) advised that it would be inappropriate for a prospective observational study conducted in primary care clinics—none of which were registered clinical trial sites and without randomized assignment—to adopt a design resembling that of a RCT. Consequently, the study design was revised to employ a site-based allocation method, wherein each institution was assigned to recruit participants for only one of the two groups.

The study protocol was approved by the Institutional Review Board of Wonkwang University Gwangju Medical Center (approval number: WKIRB-2024/10-3), and written informed consent was obtained from all participants prior to their enrollment in the study.

### 2.3. Study Registration

This study was registered with the Clinical Research Information Service (CRIS) of the Korea National Institute of Health (NIH), Republic of Korea (KCT0009932, Trial Status: Protocol version 1.2 (6 September 2024)).

### 2.4. Participating Institutions

Because this study was based on outpatient treatment settings, participating institutions were limited to sites that not only provided outpatient care but were also equipped with portable ultrasound devices.

Seven institutions were selected from among the members of the Academy of Korean Medicine Clinical Anatomy, a preliminary member society of the Association of Korean Medicine, all of which utilized portable ultrasound equipment. To ensure consistency in imaging acquisition and analysis, all participating sites used the same portable ultrasound device—AcuViz Pocket (FCU Co., Daejeon, Republic of Korea).

The list of the eight participating institutions is as follows:

1. Two outpatient departments of hospitals
  - Wonkwang University Gwangju Medical Center (Nam-gu, Gwangju Metropolitan City, Republic of Korea);
  - Jinjeop Hanyang Hospital (Namyangju, Gyeonggi Province, Republic of Korea).
2. Five outpatient departments of Korean medicine clinics
  - Kangnyung Korean Medicine Clinic (Anyang, Gyeonggi Province, Republic of Korea);
  - Gwanghwamun Kyung Hee Korean Medicine Clinic (Jongno-gu, Seoul Metropolitan City, Republic of Korea);
  - Mullae Majubom Korean Medicine Clinic (Yeongdeungpo-gu, Seoul Metropolitan City, Republic of Korea);
  - Mapo Hongik Korean Medicine Clinic (Mapo-gu, Seoul Metropolitan City, Republic of Korea);
  - Anjung Korean Medicine Clinic (Mapo-gu, Seoul Metropolitan City, Republic of Korea).

### 2.5. Participants

Participants were recruited through research information posters displayed both inside and outside each institution. Additionally, online advertisements, including blog posts, were used to supplement recruitment. As compensation, participants received the ultrasound-guided pharmacopuncture treatment at no cost.

#### 2.5.1. Inclusion Criteria

All study participants can only participate in the study if they meet all of the following criteria:

1. Adults aged 20 to 64 years;
2. Individuals who have experienced acute cervical pain within the past 7 days and are complaining of cervical pain and range of motion restrictions;
3. Individuals diagnosed with acute cervical myofascial pain syndrome at the outpatient clinic of the conducting medical institution or other medical facilities;
4. Individuals who have not received treatment for acute cervical myofascial pain syndrome at other hospitals after being diagnosed;
5. Individuals who voluntarily decide to participate in this study and sign an informed consent form.

#### 2.5.2. Exclusion Criteria

The exclusion criteria are listed below, and any individual who met at least one of these criteria was excluded from participation.

1. Individuals who have taken medication or received related treatments for muscle pain or discomfort in the neck or shoulder area that occurred within the past 7 days (including both Western and Korean medicine);
2. Individuals with a surgical history in the cervical area within the past 3 months;
3. Individuals who have experienced a sprain in the cervical area due to trauma, such as a traffic accident or contusion, within the past 3 months;
4. (For women only) Individuals who are pregnant or have given birth within the past 6 months;
5. Individuals with symptoms such as edema due to diseases of the renal endocrine system;
6. Patients currently taking anticoagulants;
7. Individuals whose cognitive function is impaired, making it difficult to understand the consent form and provide voluntary consent;
8. Individuals taking psychiatric medications due to reasons such as depression;
9. Any other individuals deemed unsuitable for participation in the study by the medical staff.

Details regarding the criteria for early termination and dropout are described in the previous study conducted by Kim et al. [8].

### 2.6. Device and Intervention

In this study, a portable ultrasound device, the AcuViz Pocket (FCU Co., Daejeon, Republic of Korea), was used to perform UGP. This device is equipped with a needle navigation system based on magnetic sensors, which allows real-time tracking of the position and direction of a magnetized needle. The system visualizes this information on the ultrasound image, thereby enhancing procedural accuracy.

The magnetized needle used in the procedure was a 26-gauge, 60 mm Acu-tracking needle (Youngchang Needle Co., Gimpo, Republic of Korea), which was designed to be removed after the injection of the pharmacopuncture solution.

HP pharmacopuncture solution was utilized as the sole pharmacological agent in this study. HP solution was prepared by the Anjung Korean Medicine Clinic Extramural Herbal Dispensary (Seoul, Republic of Korea) and is a biologically derived product obtained through the enzymatic hydrolysis of placental tissue collected from healthy donors [21]. HP solution has been reported to exhibit anti-inflammatory, analgesic, antioxidant, and tissue regenerative effects [22]. In this study, the injection was administered into the area of maximum tenderness within the SLS triangle, covering a region approximately 5 cm in diameter. A disposable 5 cc syringe (Bukwang Medical, Yangju-si, Gyeonggi-do, Republic of Korea) containing 2 cc of HP pharmacopuncture solution was utilized for each treatment [23]. The injection was performed using a 26-gauge × 60 mm guiding needle (Yongchang Co., Gimpo-si, Gyeonggi-do, Republic of Korea) under ultrasound guidance.

### 2.7. Outcome Measures

In this study, the outcome measures were established by comparing patient status immediately before and after the procedure to quantitatively assess changes in pain and functional mobility. To ensure both clinical relevance and practicality in real-world settings, pain was evaluated using the NRS, and cervical function was assessed by measuring the ROM of the cervical spine.

The NRS is a simple and sensitive tool widely used for subjective pain assessment, allowing patients to rate their perceived pain on a scale from 0 (indicating no pain) to 10 (representing the worst imaginable pain) [24].

**Figure 2:**
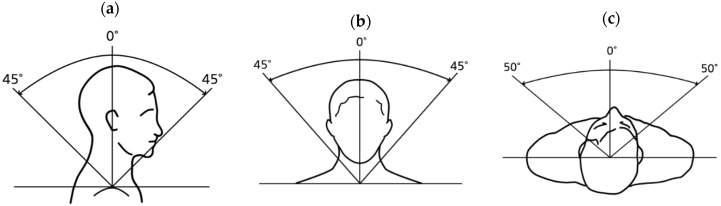
Standardized postures for measuring cervical Active Range of Motion (A-ROM): Cervical A-ROM was measured in the following directions: (**a**) flexion and extension, (**b**) lateral flexion to both sides, and (**c**) rotation to both sides. All measurements were conducted using a goniometer, with the evaluator’s line of sight and posture fixed according to the standardized guidelines provided by the Korea Occupational Safety and Health Agency. The figure illustrates the recommended postural setup and reference angles for each motion.

Cervical ROM was assessed as Active Range of Motion (A-ROM), including flexion, extension, lateral flexion to both sides, and rotation to both sides. All ROM measurements were conducted using a goniometer [25], with the evaluator’s line of sight and posture fixed in a standardized position, in accordance with the impairment evaluation guidelines of the Korea Occupational Safety and Health Agency. An illustration of the standardized measurement posture is provided in Figure 2.

Both NRS and ROM were measured using the same methods immediately before and after the procedure. We monitored adverse events through telephone follow-ups conducted immediately after treatment and again 24 to 48 h later. All assessments were performed by evaluators who had completed pre-study training and followed standardized measurement protocols. However, due to the nature of the study being conducted in a real-world clinical setting, evaluator blinding was not implemented; therefore, the possibility of measurement bias should be taken into account when interpreting the results.

### 2.8. Statistical Analysis

In this study, a two-way mixed analysis of variance (ANOVA) was conducted using the Statistical Package for the Social Sciences (SPSS) for Windows (Version 20.0; IBM Corp., Armonk, NY, USA) and RStudio (Version 2021.09.2+32; Posit Software, Boston, MA, USA) to evaluate the effects of ultrasound guidance (UGP vs. NGP) and time (pre-treatment vs. post-treatment) on participants’ pain levels (measured by the NRS) and cervical ROM. Additionally, the study aimed to examine the interaction effects between these factors. This statistical method is particularly well suited for analyzing repeated measures of dependent variables, such as NRS and ROM, and allows for the assessment of whether treatment effects differ between groups over time.

During the analysis of the study results, it was observed that, due to the characteristics of cervical myofascial pain syndrome, limitations in cervical rotation were often unilateral and predominantly occurred in a specific direction. Even in cases with bilateral limitations, patients typically exhibited more severe tenderness or reported greater pain subjectively on one side. Accordingly, NRS values were recorded based on the symptomatic side, and while ROM was measured bilaterally, the analysis of pre- and post-treatment changes focused on the affected direction for movements with lateral asymmetry. In particular, for cervical rotation and lateral flexion—where patients presented with varying directions of motion limitation—the data were standardized by aligning measurements to the affected side. These were designated as RotAff. (rotation on the affected side) and LatAff. (lateral flexion on the affected side), respectively, to enable consistent statistical comparisons.

## 3. Results

### 3.1. Baseline Characteristics

A total of 97 participants were recruited for this study. The NGP group included patients from four Korean medicine clinics, while the UGP group included patients from one Korean medicine hospital, one general hospital, and one primary clinic. Among all participants, 42 were male and 55 were female. The mean age of participants was 42.91 ± 12.94 years, and the mean BMI was 23.68 ± 3.45 kg/m^2^.

Table 1 summarizes the final number of participants recruited from each institution, and Table 2 presents the demographic characteristics of participants.

### 3.2. Clinical Characteristics of Pain Location and Movement-Induced Responses in Cervical Myofascial Pain Syndrome

#### 3.2.1. Distribution of Pain Location and Movement-Evoked Features in Patients with Cervical Myofascial Pain Syndrome

Upon clinical interviews, 33 patients reported pain exclusively in the left levator scapulae region, 32 patients reported pain exclusively in the right, and 14 patients reported bilateral pain in the levator scapulae area while in a neutral posture. Conversely, 18 patients did not report resting pain in either levator scapulae but experienced pain that was specifically reproduced during cervical rotation.

The distribution of pain location and movement-evoked features in patients with C-MPS is presented in Table 3.

#### 3.2.2. Pain Response Patterns During Cervical Rotation in Patients with Cervical Myofascial Pain Syndrome

In patients who reported pain in both levator scapulae muscles, the pharmacopuncture solution injection site was determined by the clinician based on the side with greater range of motion limitation or more severe pain.

For patients who reported pain only during cervical rotation, further assessment was conducted to determine whether there was any associated ROM restriction, as the reproduction of pain during rotation is considered a key symptom of C-MPS. Among these patients, 13 demonstrated limited ROM without pain at rest, while 38 experienced pain on both sides during cervical rotation.

The pain response patterns during cervical rotation in patients with C-MPS are summarized in Table 4.

### 3.3. Outcome Measures

#### 3.3.1. Primary Outcome

Before the intervention, the UGP group had a mean NRS score of 5.84 (standard deviation (SD) ± 2.06), and the NGP group had a mean score of 6.09 (SD ± 1.20). After the treatment, the NRS score in the UGP group decreased to 2.76 (SD ± 1.53), whereas the NGP group recorded an after-treatment score of 4.47 (SD ± 1.41).

**Figure 3:**
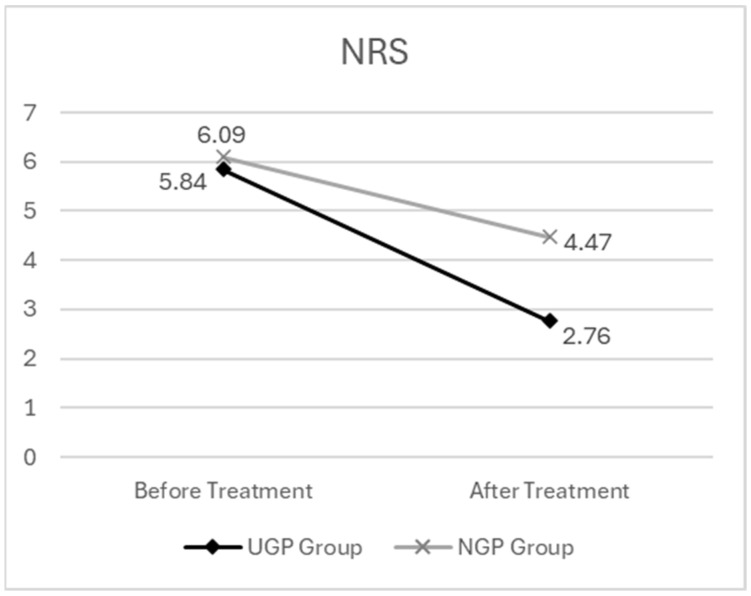
The changes in NRS scores before and after pharmacopuncture treatment.

The descriptive statistics for the NRS analysis are presented in Table 5. The graph depicting the changes in NRS scores before and after pharmacopuncture treatment is presented in Figure 3.

First, the factor of time had a statistically significant effect on NRS scores (F = 221.940, *p* < 0.001). This finding indicates that the pre-treatment NRS scores were significantly higher than the post-treatment scores across all participants, suggesting a reduction in pain levels over time.

To assess whether changes in NRS scores over time differed between groups, the interaction effect of time × group was analyzed. This interaction was found to be statistically significant (F = 21.531, *p* < 0.0001), indicating that the degree of NRS reduction over time varied between the UGP and NGP groups. Post hoc analysis revealed that while both groups showed lower NRS scores post-treatment compared to pre-treatment, the reduction in NRS scores was greater in the UGP group. This finding suggests that ultrasound-guided pharmacopuncture treatment had a meaningful effect on pain reduction among participants.

The results of the two-way mixed ANOVA for the NRS are presented in Table 6.

#### 3.3.2. Secondary Outcome

Before the intervention, the UGP group had a mean NRS score of 5.84 (standard deviation (SD) ± 2.06), and the NGP group had a mean score of 6.09 (SD ± 1.20). After the treatment, the NRS score in the UGP group decreased to 2.76 (SD ± 1.53), whereas the NGP group recorded an after-treatment.

##### ROM Flexion (Flex.)

Before treatment, the mean cervical ROM Flex. was 37.82° (SD ± 8.48) in the UGP group and 34.40° (SD ± 12.12) in the NGP group. After treatment, the UGP group showed a mean ROM of 42.99° (SD ± 8.68), whereas the NGP group showed a post-treatment ROM of 38.19° (SD ± 13.63).

**Figure 4:**
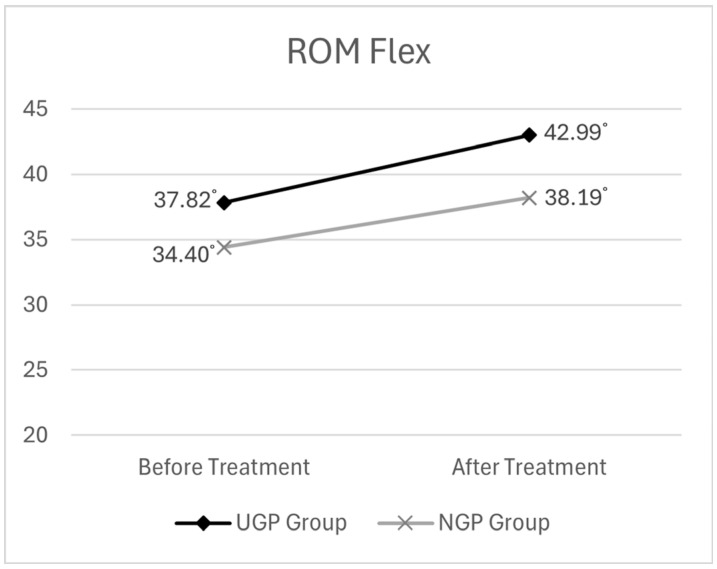
Changes in cervical Flexion ROM before and after pharmacopuncture treatment.

The descriptive statistics for the ROM Flex. analysis are presented in Table 5. The graph depicting the changes in ROM Flex. before and after pharmacopuncture treatment is presented in Figure 4.

Time was found to have a statistically significant effect on ROM Flex. (F = 37.934, *p* < 0.001). This indicates that ROM Flex scores were significantly higher after treatment compared to before, suggesting an overall improvement in range of motion over time.

However, the interaction effect of time × group was not statistically significant (F = 0.911, *p* > 0.05), indicating that the degree of ROM Flex. improvement over time did not differ between the UGP and NGP groups.

Post hoc comparisons revealed that both groups experienced an increase in ROM Flex. after the intervention, and the magnitude of change was similar between the two groups. This suggests that ultrasound guidance did not have a significant effect on the degree of improvement in cervical flexion.

The results of the two-way mixed ANOVA for ROM Flex. are presented in Table 6.

##### ROM Extension (Ext.)

Before treatment, the mean cervical ROM Ext. was 35.56° (SD ± 13.38) in the UGP group and 26.23° (SD ± 15.26) in the NGP group. After treatment, the UGP group showed a mean ROM of 44.12° (SD ± 12.19), whereas the NGP group showed a post-treatment ROM of 30.40° (SD ± 16.75).

**Figure 5:**
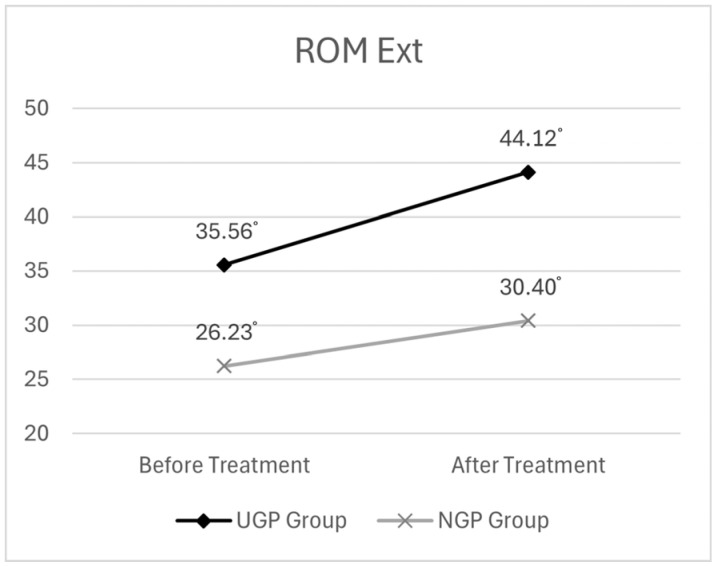
The changes in ROM of cervical extension before and after pharmacopuncture treatment.

The descriptive statistics for the ROM Ext. analysis are presented in Table 5. The graph depicting the changes in ROM Ext. before and after pharmacopuncture treatment is presented in Figure 5.

First, the analysis revealed that time had a statistically significant effect on cervical ROM Ext. (F = 93.617, *p* < 0.001), indicating that the post-treatment ROM Ext. levels were significantly higher than the pre-treatment levels across all participants, suggesting an improvement in range of motion over time.

Additionally, to assess whether the changes in ROM Ext. over time varied between groups, the interaction effect of time × group was examined and found to be statistically significant (F = 11.139, *p* < 0.01). This indicates that the degree of improvement in ROM Ext. differed between the UGP and NGP groups over time. Post hoc analysis revealed that while both groups showed increased ROM Ext. after treatment, the UGP group exhibited a greater improvement. This suggests that ultrasound-guided pharmacopuncture treatment had a significant impact on changes in ROM Ext.

The results of the two-way mixed ANOVA for ROM Ext. are presented in Table 6.

##### ROM Rotation on the Affected Side (RotAff.)

Before treatment, the mean ROM RotAff. was 47.43° (SD ± 15.19) in the UGP group and 40.72° (SD ± 26.66) in the NGP group. After treatment, the mean ROM increased to 63.04° (SD ± 15.32) in the UGP group and 46.81° (SD ± 27.69) in the NGP group.

**Figure 6:**
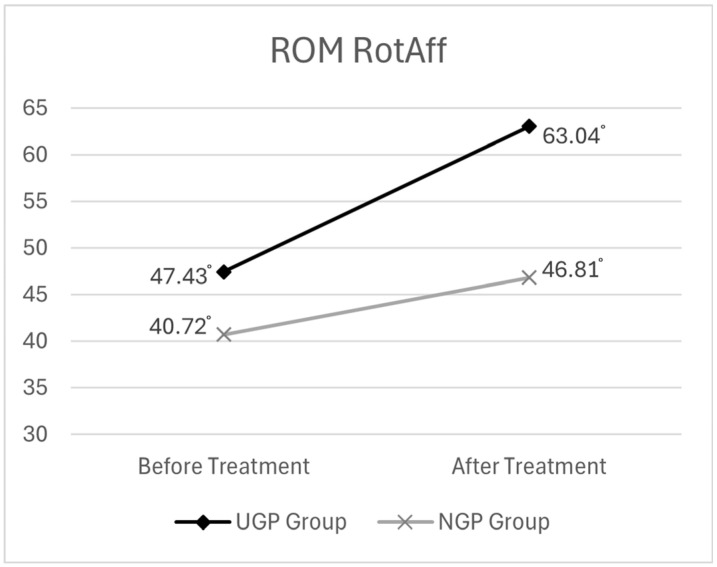
The changes in ROM of cervical rotation (on the affected side) before and after pharmacopuncture treatment.

Descriptive statistics for the ROM RotAff. analysis are presented in Table 5. The graph illustrating the changes in cervical rotation ROM before and after pharmacopuncture treatment is shown in Figure 6.

The analysis revealed that time had a statistically significant effect on ROM RotAff. (F = 95.737, *p* < 0.001). This indicates that cervical rotation ROM on the affected side significantly increased from pre- to post-treatment across all participants, suggesting an expansion in range of motion over time.

Additionally, to determine whether changes in ROM RotAff. over time differed between groups, the interaction effect of time × group was examined. The results showed a statistically significant interaction (F = 18.449, *p* < 0.001), indicating that the degree of improvement in ROM RotAff. varied between the UGP and NGP groups over time.

Post hoc analysis revealed that while both groups experienced increased ROM after treatment, the magnitude of improvement was greater in the UGP group. This suggests that ultrasound-guided pharmacopuncture treatment had a meaningful effect on enhancing cervical rotation ROM on the affected side.

The results of the two-way mixed ANOVA for ROM RotAff. are presented in Table 6.

##### ROM Lateral Flexion on the Affected Side (LatAff.)

Before treatment, the mean ROM LatAff. was 28.82° (±11.64) in the UGP group and 19.57° (±11.16) in the NGP group. After treatment, the UGP group showed an increased ROM of 36.79° (±10.45), while the NGP group recorded a post-treatment ROM of 27.32° (±10.99).

**Figure 7:**
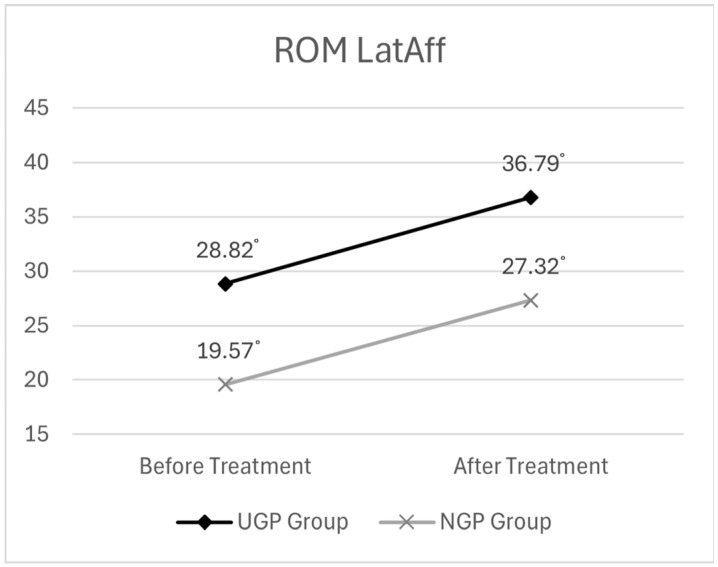
The changes in ROM of cervical lateral flexion (affected side) before and after pharmacopuncture treatment.

The descriptive statistics for ROM LatAff. are presented in Table 5, while the graphical representation of before- and after-treatment changes is illustrated in Figure 7.

The analysis revealed that time had a statistically significant effect on ROM LatAff. (F = 98.105, *p* < 0.001). This indicates that ROM LatAff. significantly increased after treatment compared to before, suggesting an improvement in cervical mobility over time.

However, when analyzing the interaction effect between time and group to assess whether the degree of ROM LatAff. improvement differed between groups, the result was not statistically significant (F = 0.019, *p* > 0.05). This indicates that the extent of ROM LatAff. improvement over time did not vary between the UGP and NGP groups—both groups demonstrated a comparable increase in cervical lateral flexion.

The results of the two-way mixed ANOVA for ROM LatAff. are presented in Table 6.

### 3.4. Safety

In this clinical study, patients were contacted via telephone immediately after the procedure and again one day later to assess safety. Among the 97 procedures performed, no adverse events were reported on the day of treatment. One patient reported headache and flu-like symptoms the following day, which were deemed unlikely to be related to the procedure.

## 4. Discussion

In this study, we confirmed that the clinical effectiveness of pharmacopuncture treatment for patients with acute C-MPS, characterized by neck and shoulder pain, varied based on the use of ultrasound guidance. The NRS scores showed a significantly greater reduction in the UGP group compared to the NGP group, indicating a more substantial alleviation of pain. This suggests that real-time ultrasound-guided targeting allows for more precise delivery of treatment to the intended anatomical structures, thereby enhancing therapeutic efficacy. These findings imply that real-time ultrasound-guided targeting provides a clinical advantage over conventional “blind” pharmacopuncture techniques.

In the cervical ROM rotation (on the affected side) and extension measures, the UGP group exhibited significantly greater improvements compared to the NGP group. This difference may be attributed to the anatomical complexity of the posterior cervical region—particularly the deep interfascial structures, such as the SLS triangle (the interfascial space formed by the intersection of the Splenius Capitis, Levator Scapulae, and Serratus Posterior Superior muscles)—which are challenging to accurately target without imaging guidance. UGP enables the precise delivery of injectates into these anatomically complex regions, thereby enhancing functional recovery.

These findings are consistent with the known pathophysiology of musculoskeletal pain. Adhesions and impaired sliding between fascial layers are recognized as major contributors to such pain, and interfascial injections can help alleviate these restrictions, restore fascial gliding, and subsequently improve both pain and function [26,27]. Additionally, it has been hypothesized that pharmacological agents act on nociceptors located within the interfascial space, modulating pain sensitivity [28]. Thus, the ability of ultrasound guidance to deliver medication accurately to these anatomical targets may have played a key role in producing the statistically significant and clinically meaningful improvements in cervical ROM observed in this study. In this context, HP pharmacopuncture—chosen for its anti-inflammatory, analgesic, and tissue-regenerative properties—may have further contributed to these effects. The targeted delivery of HP to inflamed and dysfunctional tissues through ultrasound guidance is presumed to have enhanced both pain relief and functional recovery.

In contrast, while both groups showed significant post-treatment improvements in cervical lateral flexion (on the affected side) and flexion, the differences between the groups were not statistically significant. This may be explained by the fact that the muscles surrounding the treatment site—specifically the SLS triangle—are not primarily responsible for these movements. Anatomically, cervical flexion is mainly driven by anterior muscles such as the longus colli and sternocleidomastoid, whereas lateral flexion primarily involves lateral muscles, including the sternocleidomastoid and scalenes. The musculature adjacent to the injection site in this study does not directly contribute to these specific motions. Nevertheless, the improvements observed in both groups may be linked to pain reduction in the levator scapulae region, which likely resulted in decreased muscular tension in adjacent areas, thereby facilitating smoother and more functional cervical movement overall.

In terms of safety, the advantages of UGP are clearly evident. Among a total of 97 procedures, no adverse events were reported on the day of treatment. One patient did experience mild headache and flu-like symptoms; however, according to the patient’s statement, these symptoms were attributed to an underlying cold rather than the pharmacopuncture itself, and the condition resolved spontaneously. These findings suggest that ultrasound-guided procedures may significantly reduce the risk of complications commonly associated with invasive interventions, such as bleeding, infection, or nerve damage [29]. This is particularly significant for procedures performed in anatomically sensitive areas, such as the posterior cervical region, where vascular and neural structures are densely located. The ability to visualize and avoid these critical structures under ultrasound guidance facilitates safer and more precise injection of the pharmacopuncture solution. Consequently, this supports the potential role of ultrasound guidance in enhancing procedural safety across various myofascial conditions.

In recent years, the field of Korean medicine has evolved traditional therapeutic techniques by integrating them with modern medical technology. The clinical application of ultrasound devices serves as a representative example. Ultrasound provides the advantage of real-time visualization of anatomical structures, significantly enhancing the precision and safety of invasive procedures [8,17]. Due to these characteristics, ultrasound-guided techniques have gained increasing attention, particularly in pharmacopuncture and other interventions within Korean medicine [25].

For instance, Kim Jong-Uk et al. (2019) conducted a prospective study on patients with shoulder pain and found that ultrasound-guided acupuncture (UGA) exhibited greater procedural accuracy and improved patient safety compared to non-guided procedures [20]. In addition, Lee Jin-Ho et al. conducted a systematic review and meta-analysis on the efficacy and safety of ultrasound-guided pharmacopuncture (UGP) for various musculoskeletal disorders. Their findings indicated that, compared to non-guided pharmacopuncture, UGP was significantly more effective in reducing pain and improving function, while also being associated with a lower incidence of adverse events [19]. These previous findings support the rationale for incorporating ultrasound guidance into pharmacopuncture procedures.

This study represents the first multi-center, prospective observational investigation in Korea aimed at quantitatively evaluating the clinical effectiveness and safety of UGP in patients with Cervical Myofascial Pain Syndrome (C-MPS). Conducted in real-world primary care outpatient settings and based on actual patient treatment outcomes, this research addresses the limitations of previous studies, which has largely been confined to case reports or survey-based studies with limited generalizability. In this context, the present study holds significant value as foundational research, demonstrating both clinical reproducibility and practical applicability.

In particular, the study demonstrates significant practical relevance, as it was conducted using a treatment protocol that can be realistically implemented in everyday clinical practice. The ultrasound equipment utilized was a portable device rather than a fixed model typically found in large hospitals, thereby enabling replication of the procedure in primary care clinics, including outpatient settings for Korean medicine. This highlights the feasibility of broader clinical adoption of UGP and underscores its potential for widespread application in community-based medical environments.

Moreover, the use of ultrasound imaging allowed for anatomically guided procedures that improved both precision and safety. The short-term therapeutic effects were objectively evaluated by comparing changes in pain, measured using the Numerical Rating Scale (NRS), and cervical Range of Motion (ROM) before and after treatment. Follow-up monitoring of adverse events was also conducted to evaluate the safety of the procedure.

Based on these findings, this study establishes a crucial foundation for designing high-quality clinical trials, including future randomized controlled trials (RCTs), and supports the clinical applicability of UGP as a non-surgical intervention within the Korean primary care system. Furthermore, it provides insights into the clinical significance, limitations, and future research directions regarding UGP.

This study presents the first multi-center observational evidence for the clinical use of UGP in real-world practice. However, several limitations must be acknowledged. First, the non-randomized observational design and institution-based group allocation introduce a risk of selection and performance bias. Second, the study was limited to short-term outcomes within 48 h post-treatment, providing no information on long-term efficacy. Third, interoperator variability in ultrasound proficiency may have affected the outcomes, and this was not adjusted in the analysis. Fourth, a single type of pharmacopuncture solution was used in this study, without specific standardization of its composition and concentration.

These limitations highlight important areas for improvement in future research. High-quality clinical trials with randomized allocation and blinding are needed to objectively validate the efficacy of UGP, and mid- to long-term follow-up studies are necessary to assess the durability of treatment effects. It is also important to compare the therapeutic outcomes of various pharmacopuncture solutions and evaluate how pharmacological efficacy may differ when the injectate is more precisely delivered to target tissues under ultrasound guidance. Further studies should aim to quantify differences in practitioner proficiency and explore the applicability of this intervention across a wider range of musculoskeletal conditions.

In summary, while this multi-center observational study provides preliminary clinical evidence supporting the use of UGP, it has several limitations, including its non-randomized design, short-term outcome measures, and lack of standardization in the composition of pharmacopuncture solutions. These findings highlight the need for future randomized controlled trials with extended follow-up periods and standardized protocols. Importantly, this study contributes to the foundational development of a standardized protocol for UGP, offering a valuable reference framework for future research. This framework can facilitate comparative studies of different pharmacopuncture solutions utilizing the same ultrasound-guided technique. With continued research, UGP may evolve into a precise and reliable standard intervention for musculoskeletal pain management in routine clinical practice.

## 5. Conclusions

Ultrasound-guided pharmacopuncture treatment has shown superior pain reduction and functional improvement in patients with acute cervical myofascial pain syndrome when compared to non-guided treatment. Notably, this technique may reduce risks commonly associated with invasive procedures in anatomically sensitive areas. These findings support the use of ultrasound imaging to enhance the precision, safety, and effectiveness of pharmacopuncture treatment in real-world Korean Medicine primary care settings.

## Figures and Tables

**Table 1:** Final number of participants recruited from each institution.

Institution	NGP Group	UGP Group
Wonkwang University Gwangju Medical Center	0	14
Mapo Hongik Korean Medicine Clinic	17	0
Jinjeop Hanyang Hospital	0	25
Gwanghwamun Kyung Hee Korean Medicine Clinic	0	11
Mullae Majubom Korean Medicine Clinic	17	0
Anjung Korean Medicine Clinic	3	0
Kangnyung Korean Medicine Clinic	10	0
Total	47	50

**Table 2:** Demographic characteristics of participants.

Characteristics	NGP Group	UGP Group
Gender (number (%))		
Male	21 (42%)	21 (44.68%)
Female	29 (58%)	26 (55.32%)
Age (mean (SD))	43.48 (12.89)	42.30 (13.11)
min, max, median	25, 64, 43.5	22, 64, 38
BMI (mean (SD))	23.64 (3.26)	23.72 (3.67)

**Table 3:** Distribution of pain location and movement-evoked features in patients.

Pain Pattern by Posture and Movement	Number of Patients
Left levator scapulae only (in neutral posture)	33
Right levator scapulae only (in neutral posture)	32
Bilateral levator scapulae (in neutral posture)	14
Pain reproduced during cervical rotation	18

**Table 4:** Pain response patterns during cervical rotation in patients.

Pain Response During Cervical Rotation	Number of Patients
Pain reproduced only during left rotation	23
Pain reproduced only during right rotation	23
Pain reproduced during both left and right rotation	38
No pain during cervical rotation	13

**Table 5:** Descriptive statistics.

	UGP Group		NGP Group	
	Before Treatment	After Treatment	Before Treatment	After Treatment
NRS	5.84 ± 2.06	2.76 ± 1.53	6.09 ± 1.20	4.47 ± 1.41
ROM Flex.	37.82° ± 8.48°	42.99° ± 8.68°	34.40° ± 12.12°	38.19° ± 13.63°
ROM Ext.	35.56° ± 13.38°	44.12° ± 12.19°	26.23° ± 15.26°	30.40° ± 16.75°
ROM RotAff.	47.43° ± 15.19°	63.04° ± 15.32°	40.72° ± 26.66°	46.81° ± 27.69°
ROM LatAff.	28.82° ± 11.64°	36.79° ± 10.45°	19.57° ± 11.16°	27.32° ± 10.99°

Data are presented as mean ± standard deviation (SD).

**Table 6:** Results of the two-way mixed ANOVA.

		Time	Group	Time × Group	Pairwise Comparison
NRS	F	221.940 **	11.696 *	21.531 **	UGP Group: Before > After
	P	0.000	0.001	0.000	NGP Group: Before > After
ROM Flex.	F	37.934 **	3.868	0.911	UGP Group: Before < After
	P	0.000	0.052	0.342	NGP Group: Before < After
ROM Ext.	F	93.617 **	16.213 **	11.139 *	UGP Group: Before < After
	P	0.000	0.000	0.001	NGP Group: Before < After
ROM RotAff.	F	95.737 **	7.116 *	18.449 **	UGP Group: Before < After
	P	0.000	0.009	0.000	NGP Group: Before < After
ROM LatAff.	F	98.105 **	19.779 **	0.019	UGP Group: Before < After
	P	0.000	0.000	0.889	NGP Group: Before < After

* *p* < 0.01, ** *p* < 0.001.

## Data Availability

The original contributions presented in this study are included in the article. Further inquiries can be directed to the corresponding authors.

## Abbreviations

The following abbreviations are used in this manuscript:

MPS	Myofascial Pain Syndrome
TrPs	Trigger Points
C-MPS	Cervical Myofascial Pain Syndrome
WHO	World Health Organization
DALYs	Disability-Adjusted Life Years
HIRA	Health Insurance Review and Assessment Service
UGA	Ultrasound-Guided Acupuncture
UGP	Ultrasound-Guided Pharmacopuncture
SLS	Splenius Capitis–Levator Scapulae–Serratus Posterior Superior Muscles
RCTs	Randomized Controlled Trials
SC	Splenius Capitis
LS	Levator Scapula
SPS	Serratus Posterior Superior
HP	Hominis Placenta
NGP	Non-Guided Pharmacopuncture
NRS	Numerical Rating Scale
ROM	Range of Motion
A-ROM	Active Range of Motion
ANOVA	Analysis of Variance
RotAff.	Rotation on the Affected side
LatAff.	Lateral Flexion on the Affected side
Flex.	Flexion
Ext.	Extension
